# Bayesian Adaptive Estimation with Theoretical Bound: An Exploration-Exploitation Approach

**DOI:** 10.1155/2022/1143056

**Published:** 2022-12-12

**Authors:** Mingyao Li, Juanping Zhu

**Affiliations:** School of Mathematics and Statistics, Yunnan University, Kunming, China

## Abstract

This paper investigates the theoretical bound to reduce the parameter uncertainty in Bayesian adaptive estimation for psychometric functions and proposes an exploration-exploitation (E-E) approach to improve the computation efficiency for parameter estimations. When the experimental trial goes on, the uncertainty of the parameters decreases dramatically and the space between the maximal mutual information and the theoretical bound gets narrower, so the advantage of classical Bayesian adaptive estimation algorithm diminishes. This approach tries to trade off the exploration (parameter posterior uncertainty) and the exploitation (parameter mean estimation). The experimental results show that the proposed E-E approach estimates parameters for psychometric functions with same convergence and reduces the computation time by more than 34.27%, compared with the classical Bayesian adaptive estimation.

## 1. Introduction

Bayesian adaptive estimation plays an important role in certain parameter estimations of psychometric functions [1–5]. In psychophysics, psychometric function reflects the quantitative relationship between physical stimulation and subject's psychological perception [2]. Watson and Pelli first applied the QUEST method in psychophysics [4]. Gradually, Bayesian adaptive estimation has been developed and widely used in psychophysics, behavioral and neural sciences [1, 6], clinical fields [7, 8], etc. It sequentially selects the stimulus, in the way of minimizing the uncertainty of parameters, and then updates the parameter prior distribution, to effectively estimate the parameters.

More and more practical experiments are undertaken online [9] (e.g., the research of driving behaviors [6, 10], clinical [7, 8, 11], and visual perception [12–14]). Therefore, the challenge faced by the researchers is the computation efficiency in optimizing the stimulus after collecting subject's data during typical psychophysical experiments [10]. One way is to estimate multidimensional parameters simultaneously. Kontsevich and Tyler proposed the Ψ method to estimate two-dimensional parameters [5], and Kujala and Lukka applied this method to more general psychometric functions [1, 2, 10]. Then, psychometric functions with higher dimensional parameters were estimated, such as four-dimensional parameters in the contrast sensitivity function and driving gap acceptance function [6, 13]. Furthermore, Watson extended the QUEST method to estimate psychometric parameters with multiple dimensions [15]. On the other hand, it is well known that the optimization algorithm of Bayesian adaptive estimation considers to make full use of the information contained in parameter distribution and emphasizes the convergence of the estimation. Kuss et al. discussed the importance of parameter prior distributions to extract the information contained in experimental data [3]. The authors in [16–20] considered the estimation deviation of parameters by effectively using the limited measurement information to improve the estimation efficiency.

However, in each implementation trial of Bayesian adaptive estimation, the optimization algorithm selects the most informative stimulus, by searching the parameter space of the psychometric function [1, 6, 13]. If the parameter dimension increases, the time complexity of the stimulus selection increases exponentially. The upper bound of the information gained from the optimization algorithm [21, 22] has not been well studied, and how the theoretical bound impacts the stimulus selection as well as the computation efficiency needs further investigation. Furthermore, the MSE curves of the estimated parameters in experiments usually become almost level after some trials [6], which is also desirable to be explained.

This paper investigates the theoretical upper bound of the information gain of the parameters resulting from the optimization algorithm of Bayesian adaptive estimation. This bound theoretically decides how much information the estimation algorithm can gain trial by trial and explains why the advantage of information gain from Bayesian adaptive estimation diminishes with the decrease of the uncertainty of the parameter distribution. Therefore, this paper proposes the exploration-exploitation (E-E) approach to improve classical Bayesian adaptive estimation by selecting the stimulus randomly once the low-parameter uncertainty is detected, from the perspective of machine learning [21, 23–25]. The proposed approach tries to trade off the exploration (parameter posterior uncertainty) and the exploitation (parameter mean estimation). It is not necessary for the exploitation trials to search the stimulus space and parameter space to calculate the maximal mutual information repeatedly and thus to improve the computation efficiency substantially. The proposed E-E approach is applied to two parameter estimation instances, contrast sensitivity function (CSF) and heterogeneous gap acceptance function (GAF). Experiment simulation results demonstrate that the computation time is saved by 34.74% for CSF and 34.27% for GAF with same MSE convergence. Thus, the proposed algorithm, compared to the classical Bayesian adaptive estimation, is more suitable for the practical online experiment implementations.

## 2. Problem Statement

### 2.1. Psychometric Function

In psychophysics, the psychometric function is used to describe the probability of psychological feedback after a certain stimulus is applied to the individual subject [2]. Usually, the psychometric function with multidimensional parameter *θ* ∈ *ℝ*^*l*^ is represented as Φ(*y*, *d*, *θ*), where *d* is the stimulus and *y* is the random binary feedback which indicates that the subject “rejects” or “accepts” the given stimulus. When given the stimulus *d*, the conditional probability *p*(*y*|*θ*, *d*) of the subject's feedback *y* can be expressed as

(1) $$\begin{array}{c} p\left(\left.y\right|\theta ,d\right)=\left\{\begin{array}{cc} \Phi \left(y,\theta ,d\right)\text{,} & y=1\text{,}\\ 1-\Phi \left(y,\theta ,d\right)\text{,} & y=0\text{.} \end{array}\right. \end{array}$$

The objective is to estimate the subject's true parameter *θ* of the psychometric function in as few steps as possible, due to the cost of collecting the individual subject's data. Parameter estimation problems exist in many fields such as visual [12, 13], olfactory [26, 27], and behavioral [6, 28] research.

### 2.2. Bayesian Adaptive Estimation

**Figure 1:**
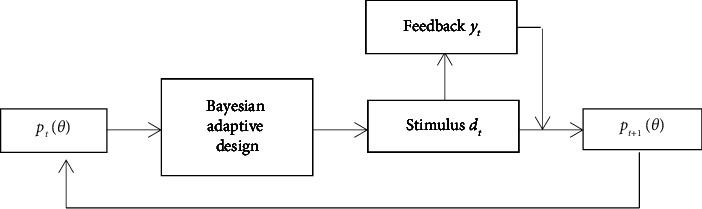
Flowchart of Bayesian adaptive estimation.

Bayesian adaptive estimation is mainly used to estimate the subject's parameter *θ* in psychometric function Φ(*y*, *d*, *θ*). Let *Y* be the random variable of the subject's feedback and Θ be the random variable of the parameters. Given the feedback space *𝒴*={0,1}, stimulus space *𝒟*⊆*ℝ*^*l*′^, and parameter space Ξ⊆*ℝ*^*l*^, it selects the most informative stimulus

(2) $$\begin{array}{c} d_{t}=\underset{d\in D}{\arg\;\max}I\left(\Theta ;Y\left|d\right.,p_{t}\left(\theta \right)\right)\text{,} \end{array}$$

where *p*_*t*_(*θ*) is the parameter prior distribution for trial *t* and mutual information *I*(Θ; *Y*|*d*, *p*_*t*_(*θ*)) measures the information gain between parameter Θ and observation *Y* of the subject [1, 10]. Observe the subject's feedback *y*_*t*_ after applying stimulus *d*_*t*_. According to Bayes rule, update the parameter posterior probability:

(3) $$\begin{array}{c} p_{t+1}\left(\theta \right)=\frac{p_{t}\left(\theta \right)p\left(y_{t}\left|\theta \right.,d_{t}\right)}{\int_{\Xi }p_{t}\left(\theta \right)p\left(y_{t}\left|\theta \right.,d_{t}\right)d\theta }\text{,} \end{array}$$

which is the prior probability for trial *t*+1. The details of Bayesian adaptive estimation algorithm can be found in [1, 5], and the flowchart is shown in Figure 1 [6].

The basic idea of Bayesian adaptive estimation is to find the most informative stimulus *d*_*t*_ to gain the maximal information in each trial *t* and thus to reduce the parameter posterior uncertainty maximally trial by trial, according to equation (3). Currently, Bayesian adaptive estimation adopts the gridding method to discretize the parameter space [29]. The parameters of psychometric function are estimated by mathematical expectation (MEAN) or maximum a posterior probability (MAP) of the parameter posterior [6, 13, 29, 30].

## 3. Theoretical Bound of Information Gain

Previous experiments indicate that the parameter posterior tends to be peaky and the uncertainty of the parameters decreases, when the implementation of Bayesian adaptive estimation converges. The parameter posterior distribution is concentrated towards the mean of the distribution. Moreover, Kujala [31] and Paninski [32] presented the asymptotic theory about the convergence of Bayesian adaptive estimation, i.e., the parameter posterior distribution is asymptotically normal [31]. Bayesian adaptive estimation selects the most informative stimulus *d*_*t*_ to gain the maximal information. However, this maximal mutual information has the upper bound, which decides that the space for the information gained from the trial *t* is limited. It is important to measure theoretically how much parameter uncertainty reduction or space for information gain can be anticipated by using this optimization strategy [1, 10, 21].

Given the psychometric function Φ(*y*, *θ*, *d*) and the conditional probability *p*(*y*|*θ*, *d*) in equation (1), the mutual information can be formulated as [1, 2, 6, 32]

(4) $$\begin{array}{c} I\left(\Theta ;Y\left|d\right.,p\left(\theta \right)\right)=\iint_{\Theta ,Y}p\left(\theta ,y\left|d\right.\right)\log\frac{p\left(\theta \left|d\right.\right)p\left(y\left|d\right.\right)}{p\left(\theta ,y\left|d\right.\right)}dyd\theta \text{,}\\ =\int_{\Xi }\int_{Y}p\left(\theta ,y\left|d\right.\right)\log\frac{p\left(\theta \left|d\right.\right)p\left(y\left|d\right.\right)}{p\left(\theta ,y\left|d\right.\right)}dyd\theta \text{.} \end{array}$$

For the symmetry, the mutual information can be rewritten as [2, 33]

(5) $$\begin{array}{c} I\left(\Theta ;Y\left|d\right.,p\left(\theta \right)\right)=I\left(Y;\Theta \left|d\right.,p\left(\theta \right)\right)=H\left(Y\left|d\right.\right)-H\left(Y\left|\Theta \right.,d\right)\\ =H\left(Y\left|d\right.\right)-\int_{\Xi }p\left(\theta \left|d\right.\right)H\left(Y\left|\theta \right.,d\right)d\theta \text{,} \end{array}$$

where

(6) $$\begin{array}{c} H\left(Y\left|d\right.\right)=h\left(p\left(y=1\left|d\right.\right)\right)=h\left(\int_{\Xi }p\left(\theta \left|d\right.\right)p\left(y=1\left|\theta \right.,d\right)d\theta \right)\text{,} \end{array}$$

(7) $$\begin{array}{c} H\left(Y\left|\theta \right.,d\right)=h\left(p\left(y=1\left|\theta \right.,d\right)\right)\text{,} \end{array}$$

where *h*(*p*)=−*p*  log*p* − (1 − *p*)log (1 − *p*) is defined as the entropy of the binary distribution with probability *p* and 1 − *p* [6].

**Theorem 1 .**

Let *d* be the stimulus and *p*(*θ*) be the prior distribution of parameter *θ*; then,

(8) $$\begin{array}{c} \max_{d\in D}I\left(\Theta ;Y\left|d\right.,p\left(\theta \right)\right)\leq H\left(\Theta \right)\text{,} \end{array}$$

where $H\left(\Theta \right)=-\underset{\theta \in \Xi }{\sum }p\left(\theta \right)\log\;p\left(\theta \right)$ is the entropy of parameter *θ*.

**Proof**

For stimulus *d*, the following holds:

(9) $$\begin{array}{c} I\left(\Theta ;Y\left|d\right.,p\left(\theta \right)\right)\leq H\left(\Theta \left|d\right.\right)\text{,} \end{array}$$

by the theory of information [31]. In fact, *p*(*θ*|*d*)=*p*(*θ*) holds given any stimulus *d*; then, *H*(Θ|*d*)=*H*(Θ). Also,

(10) $$\begin{array}{c} \max_{d\in D}I\left(\Theta ;Y\left|d\right.,p\left(\theta \right)\right)\leq H\left(\Theta \right)\text{.} \end{array}$$

**Proposition 1 .**

Let *d* be the stimulus and *Y* be the random variable of the subject's feedback; then, in Bayesian adaptive estimation,

(11) $$\begin{array}{c} H\left(\Theta \left|d\right.\right)\geq H\left(\Theta \left|Y\right.,d\right)\text{,} \end{array}$$

where *H*(Θ|*Y*, *d*) is the conditional entropy of parameter *θ*.

**Proof**

By the theory of information cannot hurt [33, 34], we can get

(12) $$\begin{array}{c} H\left(\Theta \left|d\right.\right)\geq H\left(\Theta \left|Y\right.,d\right)\text{.} \end{array}$$

Theorem 1 indicates that the mutual information *I*(Θ; *Y*|*d*, *p*(*θ*)) for any stimulus *d* will never be greater than the entropy *H*(Θ) of parameter *θ*, i.e., the information gained from the trial *t* will be less than the uncertainty of parameter *θ*, in the sequential decision of Bayesian adaptive estimation.  Proposition 1 indicates that under the observations of the random subject's feedback *Y*, the parameter entropy *H*(Θ) can be reduced for any stimulus *d* and *H*(Θ) decreases monotonically and sequentially.

In the implementation of Bayesian adaptive estimation, the parameter posterior becomes peaky and asymptotically normal [31, 32], i.e., the parameter posterior distribution is asymptotically normal such that the determinant of the posterior covariance in a certain neighborhood of the true subject parameter value is asymptotically minimal [32]. This can be explained by Proposition 1 that the uncertainty of parameters decreases monotonically. When the parameter uncertainty is low enough, the maximal mutual information will be close to the current parameter entropy. The information gained from the maximal mutual information decreases gradually, and the advantage obtained from Bayesian adaptive estimation diminishes continuously. This can clarify why the MSE curves of the estimated parameters become almost level after some trials, which is mentioned in Introduction. On the other hand, the space to reduce the parameter uncertainty is narrow and the parameter uncertainty *H*(Θ) will continuously decrease with different stimulus from Proposition 1. So, different stimuli do not create much difference in the information gain from the Bayesian inference, especially in the MSE curves of the parameters. In this case, we can use the other strategy to select the stimulus instead of the most informative stimulus without hurting the accuracy of parameter estimation.

## 4. Exploration-Exploitation Approach for Bayesian Adaptive Estimation

It should be noticed that the optimization algorithm of classical Bayesian adaptive estimation searches the parameter space and stimulus space to compute the maximal mutual information $\max_{d\in 𝒟}I\left(\Theta ;Y\left|d\right.,p_{t}\left(\theta \right)\right)$ for each trial *t*, by calling psychometric function repeatedly. According to Theorem 1, when the entropy of the parameters in the implementation of Bayesian adaptive estimation is low enough, the space to gain the information gets narrow dramatically. In this case, we can try the other strategy to select the stimulus to avoid the large computation. This paper proposes the exploration-exploitation (E-E) approach to generate the stimulus randomly to enhance the computation efficiency, instead of the most informative stimulus in Bayesian adaptive estimation, when the low-parameter entropy is detected. Therefore, this proposed approach tries to trade off the exploration (parameter posterior uncertainty) and the exploitation (parameter mean estimation).

### 4.1. Exploration Based on Maximal Mutual Information

For trial *t*, when the parameter distribution is still highly uncertain and Bayesian adaptive estimation has great advantages to explore the stimulus space, the maximal mutual information $\max_{d\in 𝒟}I\left(\Theta ;Y\left|d\right.,p_{t}\left(\theta \right)\right)$ is far away from the current bound *H*(Θ) and the algorithm chooses $d_{t}=\max_{d\in 𝒟}I\left(\Theta ;Y\left|d\right.,p_{t}\left(\theta \right)\right)$ to gain the information maximally. Then, observe the subject's response and update the parameter prior distribution by Bayesian inference.

### 4.2. Exploitation Based on Random Stimulus

For trial *t*, when the parameter distribution has low uncertainty and the maximal mutual information is close to the bound, we carry out the exploitation strategy by randomly selecting one stimulus *d*_*t*_ ∈ *𝒟*, observe the subject's response, and update the parameter prior distribution. Because no searching in the stimulus space and parameter space is required, such strategy greatly improves the computation efficiency. According to Proposition 1, this exploitation strategy will continuously reduce the parameter uncertainty *H*(Θ) and gradually sharpen the parameter distribution after Bayesian inference.

### 4.3. Algorithm for Exploration-Exploitation Approach

Based on the above analysis, we propose the algorithm of the proposed E-E approach, the exploration-exploitation Bayesian adaptive estimation. To implement the algorithm, we adopt the threshold *ε* > 0 to the bound *H*(Θ) of the maximal mutual information. If *H*(Θ) > *ε*, the proposed algorithm selects stimulus through maximal mutual information; otherwise, the algorithm selects stimulus randomly.

To estimate the parameters of given psychometric function Φ(*y*, *d*, *θ*), all inputs of Algorithm 1 are initialized. Step 2 of Algorithm 1 calculates the mutual information for all stimulus *d* in the current experimental trial. Step 3 selects the most informative stimulus, by using exploration strategy to update the parameter prior for the next trial, and calculates the parameter entropy to decide whether to go to Step 4. Step 4 selects stimulus randomly, by using exploitation strategy to update the parameter prior. The parameter estimator $\hat{\theta }$ is calculated by the MEAN of the parameter posterior.

The asymptotic theory presented by Paninski shows that the Bayesian adaptive estimation converges for psychometric functions [32]. It is well known that the convergence holds when choosing the stimulus *d*_*t*_ randomly [32]. Thus, the E-E approach will finally converge, no matter when to switch from the exploration procedure based on the maximal mutual information to exploitation procedure based on the random stimulus.

## 5. Experiment Simulations

To demonstrate the performance and computation efficiency of the proposed E-E approach for Bayesian adaptive estimation, we conduct experiment simulations for the parameter estimation problems of contrast sensitivity function (CSF) and heterogeneous gap acceptance function (GAF). The CSF and GAF are classic empirical models from the fields of vision [35] and transportation [28], respectively, and the Bayesian adaptive estimation method for CSF and GAF models was studied by Lesmes et al. [13] and Zhu and Zhang [6]. In this paper, we conduct computer simulations instead of real-word experiments. At each trial of the simulated experiment, the most informative design or random design for the parameter estimation is computed, and the subject's feedbacks are observed. The performance of the proposed EE-BAE algorithm is compared with the classical Bayesian adaptive estimation algorithm, and both algorithms are implemented in MATLAB R2018a with CPU i5-10400F, RAM (16 GB DDR4), and GPU Nvidia GeForce RTX 2060s (8G).

In Bayesian adaptive estimation, the choice of parameter initial prior distribution greatly influences the estimation convergence [2, 3, 6, 13]. This paper focuses on the performance and computation efficiency of the E-E approach with the theoretical bound. Therefore, to avoid the influence of parameter initial prior distribution, the paper adopts the non-informative uniform prior [6] as the initial prior distribution for both the proposed E-E approach and the classical Bayesian adaptive estimation. To reduce the randomness effect in the simulations, each experiment is repeated for 5000 times. In order to make fair comparisons, all initial settings and gridding settings are set the same.

The mean square error (MSE) [6, 13] between the estimated parameter value and the true value is assessed as the criterion for both algorithms. The MSE for the true parameter *θ*_ture_ in the psychometric function is defined as

(13) $$\begin{array}{c} {MSE}_{t}=\sqrt{{\left({\hat{\theta }}_{t}-\theta_{ture}\right)}^{2}}\text{,} \end{array}$$

where ${\hat{\theta }}_{t}=\underset{\theta \in \Xi }{\sum }p_{t}\left(\theta \right)\times \theta$ is the estimator of parameter *θ*_ture_ in trial *t*.

### 5.1. Contrast Sensitivity Function

Contrast sensitivity (CS) is a clinical measure to predict the functional vision. The parameter estimation problem of the contrast sensitivity function (CSF) mainly investigates how grating sensitivity varies with spatial frequency and contrast in the visual perception [13, 35]. CSF can be represented as [13, 35].

(14) $$\begin{array}{c} \Phi =\min\;\left(1-\mu ,\left(1-0.5*\left(1-10^{2\left[-S\left(f\right)-\log_{10}\left(c\right)\right]}\right)\right)\right)\text{,} \end{array}$$

where *μ*=4% and

(15) $$\begin{array}{c} S\left(f\right)=\left\{\begin{array}{cc} S^{\prime }\left(f\right)\text{,} & f\geq f_{\max}\text{,}\\ \log_{10}\left(\gamma_{\max}\right)-\delta_{1}\text{,} & f<f_{\max}\;\;and\;S^{\prime }\left(f\right)<\gamma_{\max}-\delta_{1}\text{,} \end{array}\right. \end{array}$$

with the logarithmic sensitivity

(16) $$\begin{array}{c} S^{\prime }\left(f\right)=\log_{10}\left(\gamma_{\max}\right)-\kappa {\left(\frac{\log_{10}\left(f\right)-\log_{10}\left(f_{\max}\right)}{\beta^{\prime }/2}\right)}^{2}\text{,} \end{array}$$

where *κ*=log_10_   (2), *β*′=log_10_ (2*β*_1_). *f* and *c* are the stimuli, where *f* is the grating frequency and *c* is the grating contrast. *θ*={*γ*_max_, *f*_max_, *β*_1_, *δ*_1_} is the parameter vector to be estimated. The subject has binary feedback *y* ∈ {0, 1}, where *y*=1 indicates the correct response for the grating of frequency *f* and contrast *c* and *y*=0 indicates the wrong response.

**Figure 2:**
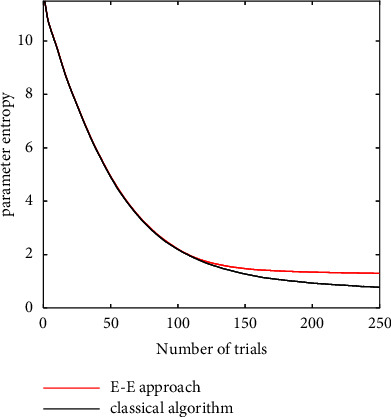
*H*_*t*_(Θ) curves of CSF. The red line is the *H*_*t*_(Θ) curve using the proposed E-E approach, and the black line is the *H*_*t*_(Θ) curve using the classical Bayesian adaptive estimation.

**Figure 3:**
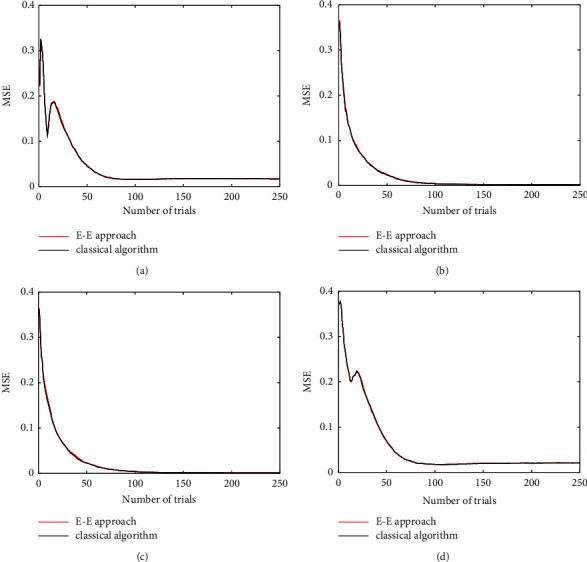
MSE curves of CSF, with the true value of parameters *θ*_ture_={100, 2.5, 2.5, 0.25}: (a) MSE of parameter *γ*_max_, (b) MSE of parameter *f*_max_, (c) MSE of parameter *β*_1_, and (d) MSE of parameter *δ*_1_. The red line is the MSE curve using the proposed E-E approach, and the black line is the MSE curve using the classical Bayesian adaptive estimation.

The ranges of the parameters in CSF are set as *γ*_max_ ∈ [2,2000], *f*_max_ ∈ [0.2,100], *β*_1_ ∈ [2,128], and *δ*_1_ ∈ [0.2,3]. The ranges of stimuli are set as *f* ∈ [log_10_ 0.2, log_10_ 36] and *c* ∈ [log_10_ 0.001, log_10_ 1] [13, 35]. The experiments are conducted by the grid searching. 20 grid points are set for each parameter, and 20 points are set for each stimulus (*f* and *c*. The parameter estimation experiment simulations are conducted for 250 trials, and the threshold of the E-E approach value in the simulations is given as *ε* = 1.5 to apply the exploitation strategy in 110 trials. The parameter entropy curves for *H*_*t*_(Θ) by the proposed E-E approach and classical Bayesian adaptive estimation can be seen in Figure 2. The MSE performances of two approaches are compared (as shown in Figure 3).

Figure 2 shows that *H*_*t*_(Θ) curves of two methods decrease monotonically as discussed in Proposition 1. Curves of the E-E approach and classical Bayesian adaptive estimation decrease quickly. This means that the proposed E-E approach can effectively reduce the uncertainty of parameters. It is reasonable to see that the red line is a little higher than the black line after the exploitation strategy is applied because classical Bayesian adaptive estimation selects the most informative stimulus for all trials.

Both the proposed E-E approach and the classical Bayesian adaptive estimation select the most informative stimulus when *H*_*t*_(Θ) > *ε*, and the E-E approach selects the stimulus randomly after *H*_*t*_(Θ) ≤ *ε*. The MSE curves of the E-E approach and the classical Bayesian adaptive estimation converge and almost overlap as shown in Figure 3. This is slightly different from the *H*_*t*_(Θ) curves because we take the mean to compute the parameter estimator. Therefore, the proposed E-E approach trades off the parameter posterior uncertainty and the parameter mean estimation.

Figure 3 shows that both the proposed E-E approach and the classical Bayesian adaptive estimation can accurately estimate all parameters in the CSF estimation, and the difference between their MSE performance is marginal. For 250 trials, the experiment of the E-E approach runs for 7.57 seconds, but the classical Bayesian adaptive estimation runs for 11.60 seconds. The computation time of the E-E approach is substantially shortened by 34.74%.

### 5.2. Heterogeneous Gap Acceptance Function

The heterogeneous gap acceptance studies the driver's response (acceptance or rejection) to different driving gaps when crossing a traffic stream, to provide the driving propensity of the individual driver [6, 28]. Miller's heterogeneous gap acceptance function with two parameters is represented as [6, 28].

(17) $$\begin{array}{c} \Phi =\Phi \left(\frac{d-{\bar{T}}_{cr}}{\sigma }\right)\text{.} \end{array}$$

The stimulus *d* is the gap which driver faces, and Φ(∙) is the standard cumulative normal probability function. Binary feedback *y*=1 indicates that the driver accepts the gap *d* when facing it, and *y*=0 indicates that the driver rejects *d*. $\theta =\left\{{\bar{T}}_{cr},\sigma \right\}$ are the driver's parameters to be estimated.

**Figure 4:**
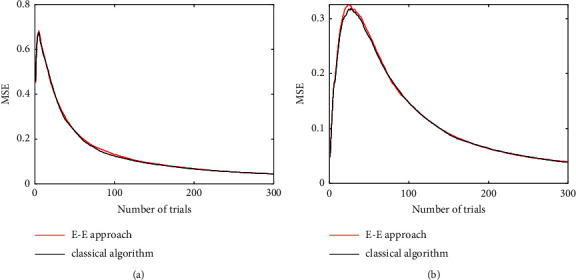
MSE curves of heterogeneous gap acceptance function, with the true parameter *θ*_ture_={7.3, 2.2804}: (a) MSE of parameter ${\bar{T}}_{cr}$; (b) MSE of parameter *σ*. The red line is the MSE curve using the proposed E-E approach, and the black line is the MSE curve using the classical Bayesian adaptive estimation.

**Figure 5:**
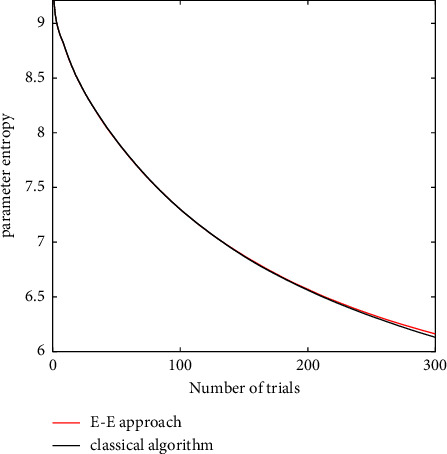
*H*_*t*_(Θ) curves of GAF. The red line is the *H*_*t*_(Θ) curve using the proposed E-E approach, and the black line is the *H*_*t*_(Θ) curve using the classical Bayesian adaptive estimation.

The ranges of the parameters of GAF are set as ${\bar{T}}_{cr}\in \left[5,10\right]$, *σ* ∈ [1,4]. The range of stimuli *d* is set as *d* ∈ [4,12] [6, 28]. The experiment simulations are conducted by the grid searching. 20 grid points are set for each parameter, and 25 points are set for the stimulus *d*. The experiments are taken for 300 trials, and the threshold of the E-E approach is set as *ε* = 6.4 to apply the exploitation strategy in 160 trials. The parameter uncertainty is compared in each experimental trial by the E-E approach and classical Bayesian adaptive estimation, as shown in Figure 4. The MSE comparisons for GAF parameter estimation by two methods are shown in Figure 5.

Figure 5shows that *H*_*t*_(Θ) in the parameter entropy of CSF between two methods decreases monotonically and quickly. Similar to the explanation for the results of CSF, the red E-E approach line diverts a little higher from the black classical algorithm line.

Figure 4 shows the performance comparisons of the parameter estimation of GAF between the E-E approach and the classical Bayesian adaptive estimation. Similar to the results of CSF experiments, the MSE curves of both E-E approach and classical Bayesian adaptive estimation converge and the MSE difference between two methods is minor (as shown in Figure 4). The parameter estimation of ${\bar{T}}_{cr}$ converges faster than parameter *σ* obviously. GAF has two parameters, and CSF has four parameters to be estimated. The true parameter values for CSF are selected far away from the mean of initial prior distribution, and the true parameter values for GAF are selected close to the mean of initial prior distribution. So, the shapes of MSE curves of GAF are different from CSF, and the convergence of the estimations takes more trials. For 300 trials, the experiment of the E-E approach runs for 2.11 seconds, but the classical Bayesian adaptive estimation takes 3.21 seconds. The computation time of the E-E approach is saved by 34.27%.

## 6. Conclusion

The paper investigates the theoretical bound of the information gained from Bayesian adaptive estimation for the parameter estimation in psychometric functions. The advantage to gain the information from classical Bayesian adaptive estimation is limited when the parameter posterior distribution gets peaky. Especially, the bound of the information gain gradually decreases when the estimation experimental trial goes on. Thus, the paper proposes the exploration-exploitation approach to accelerate the computation by selecting the stimulus randomly once the low-parameter uncertainty is detected and trades off the parameter posterior uncertainty and the parameter mean estimation. The experiment simulation results, from the parameter estimations of psychometric functions CSF and GAF, indicate that the proposed approach improves the computation efficiency by 34.74% for CSF and 34.27% for GAF with the same accuracy for estimations. This computation efficiency is well suitable for online experiments. The proposed exploration-exploitation approach for Bayesian adaptive estimation can be applied in parameter estimations of various psychometric functions in psychophysics. It can be also extended to behavioral and neural sciences and clinical and more fields using the idea of Bayesian adaptive estimation.

## Figures and Tables

**Algorithm 1:**
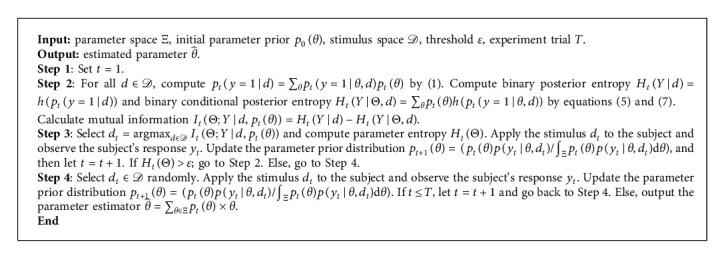
Exploration-exploitation Bayesian adaptive estimation (EE-BAE).

## Data Availability

No underlying data were collected or produced in this study.
